# TRPV6 Determines the Effect of Vitamin D3 on Prostate Cancer Cell Growth

**DOI:** 10.1371/journal.pone.0016856

**Published:** 2011-02-11

**Authors:** V'yacheslav Lehen'kyi, Maylis Raphaël, Agathe Oulidi, Matthieu Flourakis, Sergii Khalimonchyk, Artem Kondratskyi, Dmitri V. Gordienko, Brigitte Mauroy, Jean-Lois Bonnal, Roman Skryma, Natalia Prevarskaya

**Affiliations:** 1 Inserm, U-1003, Equipe labellisée par la Ligue Nationale contre le cancer, Villeneuve d'Ascq, France; 2 Université des Sciences et Technologies de Lille (USTL), Villeneuve d'Ascq, France; 3 Division of Basic Medical Sciences, St. George's University of London, Cranmer Terrace, London, United Kingdom; 4 Université Catholique de Lille, Service d'Urologie, Lille, France; University of Medicine and Dentistry of New Jersey, United States of America

## Abstract

Despite remarkable advances in the therapy and prevention of prostate cancer it is still the second cause of death from cancer in industrialized countries. Many therapies initially shown to be beneficial for the patients were abandoned due to the high drug resistance and the evolution rate of the tumors. One of the prospective therapeutical agents even used in the first stage clinical trials, 1,25-dihydroxyvitamin D3, was shown to be either unpredictable or inefficient in many cases. We have already shown that TRPV6 calcium channel, which is the direct target of 1,25-dihydroxyvitamin D3 receptor, positively controls prostate cancer proliferation and apoptosis resistance (*Lehen'kyi et al.*, Oncogene, 2007). However, how the known 1,25-dihydroxyvitamin D3 antiproliferative effects may be compatible with the upregulation of pro-oncogenic TRPV6 channel remains a mystery. Here we demonstrate that in low steroid conditions 1,25-dihydroxyvitamin D3 upregulates the expression of TRPV6, enchances the proliferation by increasing the number of cells entering into S-phase. We show that these pro-proliferative effects of 1,25-dihydroxyvitamin D3 are directly mediated via the overexpression of TRPV6 channel which increases calcium uptake into LNCaP cells. The apoptosis resistance of androgen-dependent LNCaP cells conferred by TRPV6 channel is drastically inversed when 1,25-dihydroxyvitamin D3 effects were combined with the successful TRPV6 knockdown. In addition, the use of androgen-deficient DU-145 and androgen-insensitive LNCaP C4-2 cell lines allowed to suggest that the ability of 1,25-dihydroxyvitamin D3 to induce the expression of TRPV6 channel is a crucial determinant of the success or failure of 1,25-dihydroxyvitamin D3-based therapies.

## Introduction

Prostate cancer remains the most common noncutaneous human malignancy and the second most lethal tumor among men with the highest incidence in industrialized countries [1]. The androgen receptor and other steroids regulate vital aspects of prostate cellular growth and function including proliferation, differentiation, apoptosis, lipid metabolism, and secretory action [2]. Androgen suppression has been the leading treatment and currently the most successful [3]. However, prostate carcinomas eventually become androgen-irresponsive, and the cancer is refractory to hormonal therapy — the most important reason for prostate cancer mortality [4].

Different nuclear receptors have been targeted for therapy and among them 1,25-dihydroxyvitamin D3 which exerts a multitude of anti-tumor activities against cultured prostate cancer cells and xenografts [5]. Normal and malignant prostatic epithelial cells express vitamin D3 receptor (VDR), and activation of VDR by 1,25-dihydroxyvitamin D3 generally results in inhibition of proliferation and cell cycle arrest [6]. However, to prevent or treat prostate cancer, the interactions of other nuclear receptors and signaling pathway need to be considered [7].

The function of ion channels has been discussed in relation to proliferation and apoptosis. More recently, store operated Ca^2+^ channels and the Ca^2+^ pool in the endoplasmatic reticulum have also been related to prostate cancer development [8]. Proliferation of the prostate cancer cell lines LNCaP and PC3 was inhibited by TH-1177, a substance which blocks Ca^2+^ entry [9]. Alterations in Ca^2+^ pool and cytosolic Ca^2+^ have not only been described to increase proliferation and sarcoendoplasmatic Ca^2+^-ATPase (SERCA) expression in LNCaP cells [10], but also to induce apoptosis [11]. Thus, Ca^2+^ homeostasis is critically involved in cancer development and progression.

Our attention has been drawn by the observation that a transient receptor potential highly Ca^2+^-selective channel subfamily V member 6, TRPV6 is strongly expressed in advanced prostate cancer and significantly correlates with the Gleason >7 grading representing a strong marker of tumor progression and subsequent invasion into the healthy tissues [12], [13]. We have previously shown that TRPV6 forms highly calcium selective channels in prostate cells, whose current amplitude and inactivation behavior are tightly regulated by the intracellular calcium concentration [10], [14]. Besides we have already shown that TRPV6 channel is involved in the control of prostate cancer proliferation and apoptosis resistance [15]. However, the precise role of TRPV6 in prostate pathophysiology remains illusive, and its regulation by androgen – contradictive [16]. Moreover, VDR being a direct activator of *trpv6* promoter [17], and 1,25-dihydroxyvitamin D3 a widely used anticancer treatment have completed an intriguing hypothesis for TRPV6 regulation and significance in prostate cancer. Our studies were based on the fact that 1,25-dihydroxyvitamin D3, already used in the first stage of clinical trials was shown to be either unpredictable or inefficient in many cases, and the fact that TRPV6 which positively controls prostate cancer proliferation and apoptosis resistance [15] is a direct target of 1,25-dihydroxyvitamin D3 [17]. The question how the known 1,25-dihydroxyvitamin D3 antiproliferative effects may be compatible with the upregulation of pro-oncogenic TRPV6 channel was the aim of our study.

## Materials and Methods

### Cell culture

Human LNCaP (lymph node cancer of the prostate), LNCaP C4-2, and DU-145 cell lines were obtained from American Type Culture Collection (ATCC) and cultured in RPMI medium (Gibco-BRL, CergyPontoise, France) supplemented with 10 or 2% foetal calf serum (FCS) and containing kanamycin (100 µg/ml) and l-glutamine (2 mM). Cells were cultured at 37°C in a humidified atmosphere with 5% CO_2_ in air. The medium was changed three times a week and cultures were split by treating the cells with 0.25% trypsin (in PBS) for 5 min at 37°C before reaching confluency. For the experiments, cells were seeded in 6-well plates for PCR and western-blotting and onto glass coverslips for immunocytochemistry and calcium imaging. For the 1,25-dihydroxyvitamin D3 studies cells were treated with EtOH as a control for 1,25-dihydroxyvitamin D3. Charcoal-striped foetal calf serum (2%) was added to phenol red free RPMI medium together with kanamycin and L-glutamin as above to incubate the cells to create steroid-deprived conditions.

### RT- PCR

Total RNA was isolated using the guanidium thiocyanate-phenol-chloroform extraction procedure. After DNase I (Life Technologies) treatment to eliminate genomic DNA, 2 µg of total RNA was reverse transcribed into cDNA at 42°C using random hexamer primers (Perkin Elmer) and MuLV reverse transcriptase (Perkin Elmer) in a 40 µl final volume, followed by real time quantitative PCR.

### Quantitative real-time PCR

Quantitative real-time PCR of TRPV6 and HPRT mRNA transcripts was done using MESA GREEN qPCR MasterMix Plus for SYBR Assay (Eurogentec, France) on the Biorad CFX96 Real-Time PCR Detection System. The sequences of primers are indicated in Table 1. The HPRT gene was used as an endogenous control to normalize variations in RNA extractions, the degree of RNA degradation, and variability in RT efficiency. To quantify the results we used the comparative threshold cycle method ΔΔC(t).

**Table 1:** Primers and siRNA.

No	Name, Accession <$>\raster="rg1"<$>	Forward(5′-…- 3′)	Backward(5′-…- 3′)	Expected Size (b.p)
1.	TRPV6, NM_018646	TTGGCAGCTAGAAGGAGAGG	TCTGCAGATGGTTCCAGAGA	106
2.	HPRT, (NM_000194)	GGCGTCGTGATTAGTGATGAT	CGAGCAAGACGTTCAGTCCT	134
3.	TRPV6siRNA	5′-CCUGCUGCAGCAGAAGAGG(dTdT)-3′		
4.	TRPV6siRNA-1	5′-GACTCTCTATGACCTCACA(dTdT)-3′		
5.	AR, siRNA	5′-GACUCAGCUGCCCCAUCCA(dTdT)-3′		

### Western-blotting

Semiconfluent LNCaP cells were treated with an ice-cold lysis buffer containing: 10 mM Tris-HCl, pH 7.4, 150 mM NaCl, 10 mM MgCl, 1 mM PMSF, 1% Nonidet P-40, and protease inhibitor cocktail from Sigma. The lysates were centrifuged 15,000× g at 4°C for 20 minutes, mixed with a sample buffer containing: 125 mM Tris-HCl pH 6.8, 4% SDS, 5% β-mercaptoethanol, 20% glycerol, 0.01% bromphenol blue, and boiled for 5 min at 95°C. Total protein samples were subjected to 8, 10, and 15% SDS-PAGE and transferred to a nitrocellulose membrane by semi-dry Western blotting (Bio-Rad Laboratories). The membrane was blocked in a 5% milk containing TNT buffer (Tris-HCl, pH 7.5, 140 mM NaCl, and 0.05% Tween 20) overnight then probed using specific rabit polyclonal anti TRPV6 antibody (Alomone Labs Ltd., 1/200), anti-PCNA (Santa-Cruz, 1/1000), anti-β-actin (Lab Vision Co., 1/1000) antibodies. The bands on the membrane were visualized using enhanced chemiluminescence method (Pierce Biotechnologies Inc.). Densitometric analysis was performed using a Bio-Rad image acquisition system (Bio-Rad Laboratories).

### Immunocytochemistry

The cells grown on the glass coverslips were washed once with PBS and, if appropriate, incubated with Cholera toxin subunit B Alexa Fluor® 488 conjugate (Molecular Probes, 1/2000) for 15 min, then washed once with PBS and fixed in 3.5% paraformaldehyde in PBS. PBS-glycine (30 mM) was used to quench the reaction with the subsequent permeabilization with 0.1% Triton X-100. The cells were washed again in PBS and subjected to conventional immunostaining procedure. Alexa Fluor® 546 goat anti-rabbit IgG (Molecular Probes, 1/4000) was used as a secondary antibody for TRPV6 staining. Fluorescence analysis was carried out using Carl Zeiss Laser Scanning Systems LSM 510 connected to a Zeiss Axiovert 200 M with 63×1.4 numerical aperture oil immersion lens at room temperature. Both channels were excited, collected separately and then merged using software Carl Zeiss LSM Image Examiner.

### Cell proliferation

Cell proliferation was measured using the CellTiter 96 Aqueous One Solution cell proliferation assay (Promega, Madison, WI), on the basis of the cellular conversion of the colorimetric reagent MTS [3,4-(5-dimethylthiazol-2-yl)-5-(3-carboxymethoxyphenyl)-2-(4-sulfophenyl)-2H-tetrazolium salt] into soluble formazan by dehydrogenase enzymes found only in metabolically active, proliferating cells. Following each treatment, 20 µl of dye solution was added into each well in 96-well plate and incubated for 2 h. Subsequently, absorbance was recorded at 490 nm wavelength using an ELISA plate reader (Molecular Devices, Sunnyvale, CA). Cellular proliferation inhibition rate is calculated as: (*A*_control_-*A*_sample_)/(*A*_control_-*A*_blank_)×100%.

### Cell cycle and apoptosis assays

Flow cytometry assays were performed on cell populations cultured in triplicate 25-cm^2^ flasks as originally described [18]. Approximately 10^6^ cells were fixed with 1 ml ice-cold 70% methanol for 30 min. After fixing, cells were pelleted by centrifugation to remove the fixatives, washed three times with phosphate-buffered saline (PBS) at 4°C, resuspended in 100 µl PBS, treated with 100 µl RNAse A (1 mg/ml, Sigma), and stained with propidium iodide (PI, Sigma) at a final concentration of 50 µg/ml. The stained cells were stored at 4°C in the dark and analyzed within 2 h. The stained samples were measured on a FACScan flow cytometer (Becton–Dickinson, San Jose, CA). Data were acquired for 7000 events with a variation coefficient of less than 5%, and red fluorescence was measured using a fluorescence detector 3 (FL3) on the *X*-axis. The data were stored and analyzed using CellQuest software to assess cell-cycle distribution patterns (subG1 (apoptotic), G0/G1, S, and G2/M phases).

### Calcium Imaging

Cells were plated onto glass coverslips and were loaded with 4 µM Fura-2 AM at room temperature for 45 min in the growth medium. Recordings were performed in HBSS containing (in mM): 140 NaCl, 5 KCl, 2 MgCl_2_, 0.3 Na_2_HPO_3_, 0.4 KH_2_PO_4_, 4 NaHCO_3_, 5 glucose and 10 HEPES adjusted to pH 7.4 with NaOH. CaCl_2_ was adjusted to 0.07 mM or 1,8 mM depending on the experiment. The coverslips were then placed in a perfusion chamber on the stage of the microscope. Fluorescence images of the cells were recorded with a video image analysis system (Quanticell). The Fura-2 fluorescence, at the emission wavelength of 510 nm, was recorded by exciting the probe alternatively at 340 and 380 nm. The signal ratio at 340/380 nm was converted into [Ca^2+^]_i_ level using an *in vitro* calibration.

### siRNA cell transfection

LNCaP cells were transfected overnight with 200 nM of siRNA-TRPV6 1 and 2 per well of a six-well plate using “Gene porter 2” (Gene Therapy Systems, Inc.) in a final volume of 1 ml. Ready-to-use siRNA-TRPV6s (processing option:A4) were synthesized by Dharmacon Research Inc (Lafayette, USA)(see Table 1).

### Reagents

All reagents were purchased from Sigma (Sigma, L'Isle d'Abeau Chesnes, France) unless otherwise specified.

### Statistics

Data were expressed as mean±SD. Statistical analysis were carried out using Student's unpaired *t*-tests. * - P<0.05 or ** - P<0.01 indicate statistical significance.

## Results

**Figure 1:**
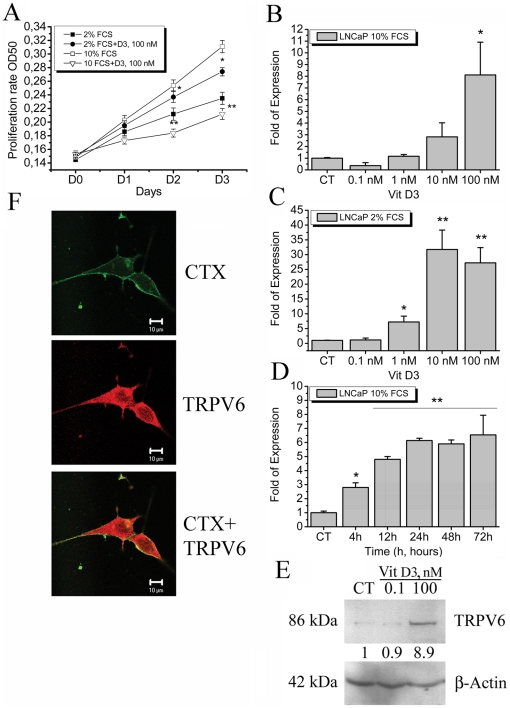
The effects of 1,25-dihydroxyvitamin D3 on proliferation of LNCaP cells and expression of TRPV6 channel. **A**, 1,25-dihydroxyvitamin D3 effects on proliferation rate measured by MTS assay of LNCaP cells incubated either with 2% or 10% FCS-containing RPMI medium, * - P<0.05, ** - P<0.01, as compared to their respective controls (DMSO), n = 3. **B**, The upregulation of TRPV6 mRNA expression by 1,25-dihydroxyvitamin D3 in LNCaP cells cultured in 10% FCS-containing RPMI medium; * - P<0.05, as compared to control (DMSO), n = 3. **C**, The upregulation of TRPV6 mRNA expression by 1,25-dihydroxyvitamin D3 in LNCaP cells cultured in 2% FCS-containing RPMI medium; * - P<0.05, ** - P<0.01, as compared to control (DMSO), n = 3. **D**, The time-dependence of TRPV6 expression under 100 µM 1,25-dihydroxyvitamin D3 treatment in LNCaP cells incubated in 10% FCS-containing RPMI medium. * - P<0.05, ** - P<0.01, as compared to control (DMSO), n = 3. **E**, a western-blotting of TRPV6 protein levels induced by 1,25-dihydroxyvitamin D3 treatment for 3 days in LNCaP cells incubated in 2% FCS-containing RPMI medium. **F**, A confocal microscopy showing the pattern of TRPV6 protein expression and localisation onto the plasma membrane of LNCaP cells cultivated in 2% FCS-containing RPMI medium. Cholera toxin conjugated to FITC (CTX, green) used to stain the plasma membrane as well as the TRPV6 channel (TRPV6, red), and their respective merge (CTX+TRPV6) are shown.

The effect of 1,25-dihydroxyvitamin D3 on prostate cancer cell proliferation has been studied in two experimental conditions: 2% and 10% foetal calf serum (FCS)-supplemented RPMI medium. The growth of androgen-dependent LNCaP cell line was surprisingly increased by 100 nM 1,25-dihydroxyvitamin D3 in 2% FCS supplemented medium and suppressed in 10% FCS (Fig. 1A). We have already demonstrated the role of TRPV6 channel in proliferation of prostate cancer cells [15], and therefore we sought to investigate the regulation of TRPV6 channel expression by 1,25-dihydroxyvitamin D3. Since it has been shown that *trpv6* is a VDR-regulated gene [17], we have studied the regulation of TRPV6 expression by 1,25-dihydroxyvitamin D3 in LNCaP cells in different steroid content of the media (Fig. 1B, C). 1,25-dihydroxyvitamin D3 appears to directly activate the *trpv6* gene in LNCaP cells, though in 10% FCS medium its effects were not that significant (Fig. 1B) than in 2% FCS (Fig. 1C). 1,25-dihydroxyvitamin D3 significantly dose-dependently increased TRPV6 mRNA expression in 2% FCS-containing RPMI medium (Fig. 1C). To check whether the diminished effects of 1,25-dihydroxyvitamin D3 were due to FCS content and not to the optimal effect time we performed the time curve using the maximal concentration of 100 nM over three days at different time intervals (Fig. 1D). To confirm the significant induction of TRPV6 protein by 1,25-dihydroxyvitamin D3 in 2% FCS containing RPMI medium obtained by real time quantitative PCR a western-blotting was performed. It showed a considerable increase in TRPV6 protein level upon activation with 100 nM 1,25-dihydroxyvitamin D3 (Fig. 1E). Immunocytochemistry using TRPV6 specific antibody showed the expression of TRPV6 channels in LNCaP cells (Fig. 1F) as well as its localisation on the plasma membrane using Cholera toxin (CTX) conjugated with FITC labelling specifically G2M lipids in the membrane. Hence, the effects of 1,25-dihydroxyvitamin D3 on the growth of androgen-dependent LNCaP cells depend on the relative steroid content. Besides, 1,25-dihydroxyvitamin D3 significantly increases the expression of TRPV6 channel in low-steroid conditions.

### TRPV6 is involved in1,25-dihydroxyvitamin D3-induced proliferation of LNCaP cells

**Figure 2:**
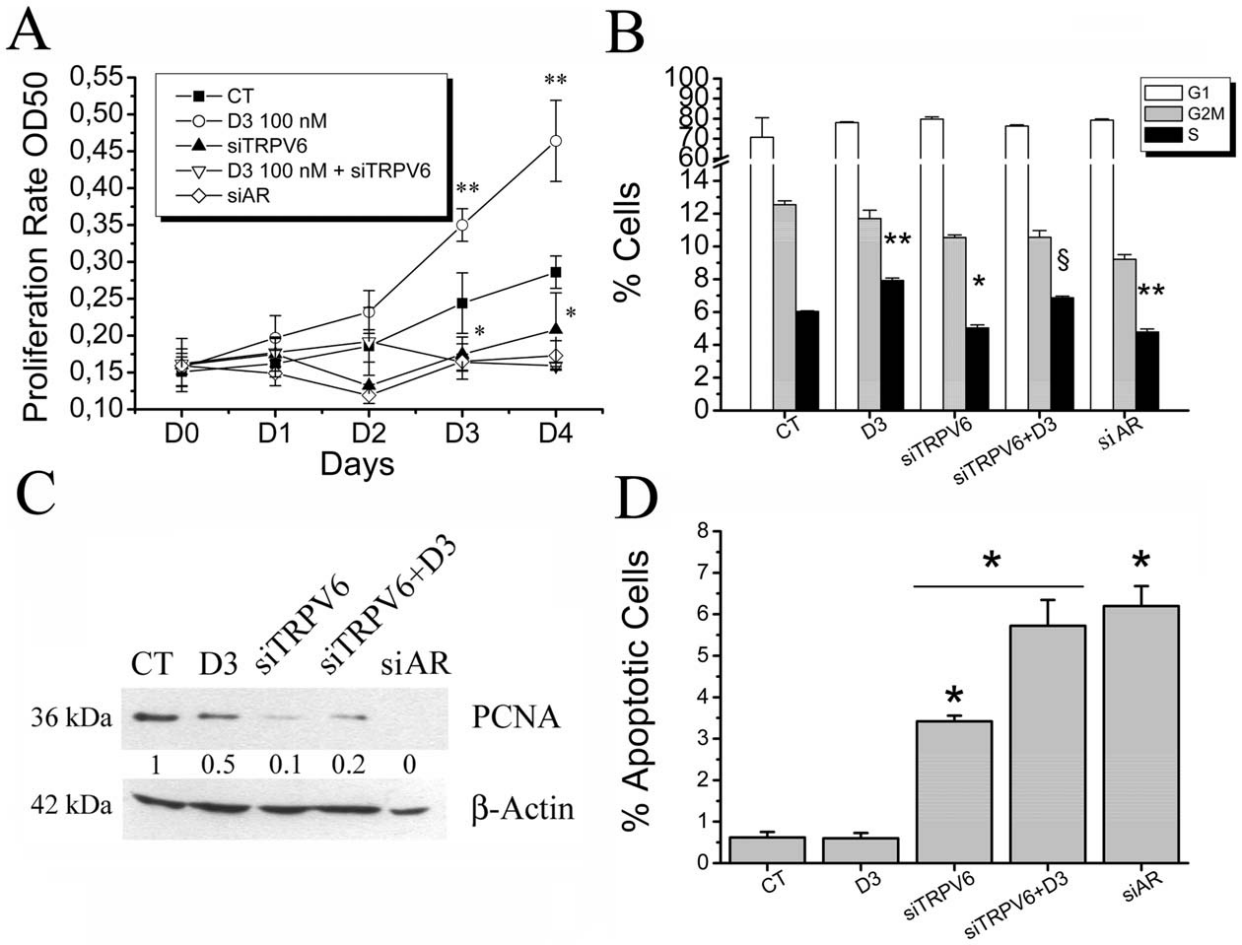
The effects of 1,25-dihydroxyvitamin D3 on proliferation and apoptosis resistance of LNCaP cells are mediated via TRPV6 channel. **A**, LNCaP cells proliferation in 2% FCS-containing RPMI medium treated with 1,25-dihydroxyvitamin D3 (100 nM, applied at D1), siRNA-TRPV6 (siTRPV6, 80 nM, transfected at D0), the combined treatment of 1,25-dihydroxyvitamin D3 and siTRPV6 specified above, and siRNA-AR (siAR, 80 nM, transfected at D0) as a positive control. * - P<0.05, ** - P<0.01, as compared to control, n = 4; **B**, a cell cycle assay of LNCaP cells (incubated with 2% FCS-containing RPMI medium) for the same conditions as in MTS assay (**A**) (D3 equals 100 nM 1,25-dihydroxyvitamin D3), carried out by flow cytometry of the cells stained with propidium iodide. * - P<0.05, ** - P<0.01, § - P<0.05 vs. Vitamin D3; n = 3. **C**, a western-blotting of proliferating cell nuclear antigen (PCNA) in the conditions indicated above as compared to β-actin. **D**, an apoptosis assay carried out by flow cytometry as a subG1 population of LNCaP cells cultured in 2% FCS-containing RPMI medium stained with propidium iodide. * - P<0.01 vs. control; n = 3.

According to the data obtained above the effects of 1,25-dihydroxyvitamin D3 in 2% FCS were further studied. Since we have already demonstrated the role of TRPV6 channel in proliferation of prostate cancer cells [15], and knowing that there is no chemical compound available so far to selectively block TRPV6, we used siRNA approach to selectively knockdown TRPV6. Three different methodological approaches were employed to assess proliferation of LNCaP cells in 2% FCS-containing medium (Fig. 2A–C). The number of viable proliferating cells was measured by MTS assay. siRNA-TRPV6 significantly decreased the number of proliferating cells from day 2 to 4 after transfection (D0) (Fig. 2A). 100 nM 1,25-dihydroxyvitamin D3 was able to increase proliferation of LNCaP cells whereas TRPV6 knockdown inversed this stimulation to the level even lower than in control. siRNA against androgen receptor (AR), known to be crucial for prostate growth and development, was used as a positive control to achieve strong and reliable effects on prostate cell viability.

A cell cycle assay using propidium iodide staining was performed to precise the effects of TRPV6 knockdown as well as 1,25-dihydroxyvitamin D3 effects and the role of TRPV6 therein, on cell cycle phase distribution of LNCaP cells cultured in 2% FCS containing RPMI medium (Fig. 2B). Indeed, we confirmed that siRNA-TRPV6 decreased the number of cells entered into the S-phase. The percentage of the cells entered into the S-phase was significantly higher in 100 nM 1,25-dihydroxyvitamin D3 treated cells than in control. Pretransfection of LNCaP cells with siRNA-TRPV6 attenuated 1,25-dihydroxyvitamin D3 increased proliferation, though not to the full extent. siRNA-AR as above was used as a positive control and showed a considerable decrease in % of the cells entered into the S-phase.

We also monitored a protein level of proliferating cell nuclear antigen (PCNA) using the same conditions. PCNA appeared to be significantly decreased upon siRNA-TRPV6 knockdown. 1,25-dihydroxyvitamin D3-treated cells expressed 2-fold less PCNA as was also observed by the combined treatment of siRNA-TRPV6 and 100 nM 1,25-dihydroxyvitamin D3. The level of PCNA in siRNA-AR-treated cells was undetectable (Fig. 2C).

A cell cycle assay also allowing measuring a number of apoptotic cells as a subG1 population was employed. 100 nM 1,25-dihydroxyvitamin D3 had no influence on apoptosis itself, whereas siRNA-TRPV6 had significant effect on apoptosis rate (Fig. 2D). However, combining the treatment of 100 nM 1,25-dihydroxyvitamin D3 with the transfection of siRNA-TRPV6 significantly increased the number of apoptotic cells much more than siRNA-TRPV6 pretreatment alone (Fig. 2D). Thus, TRPV6 is involved in both proliferation and apoptosis resistance of LNCaP cells and the effects of 1,25-dihydroxyvitamin D3 are strongly dependent on TRPV6 expression.

### TRPV6 mediates 1,25-dihydroxyvitamin D3-induced Ca^2+^-uptake in LNCaP cells

**Figure 3:**
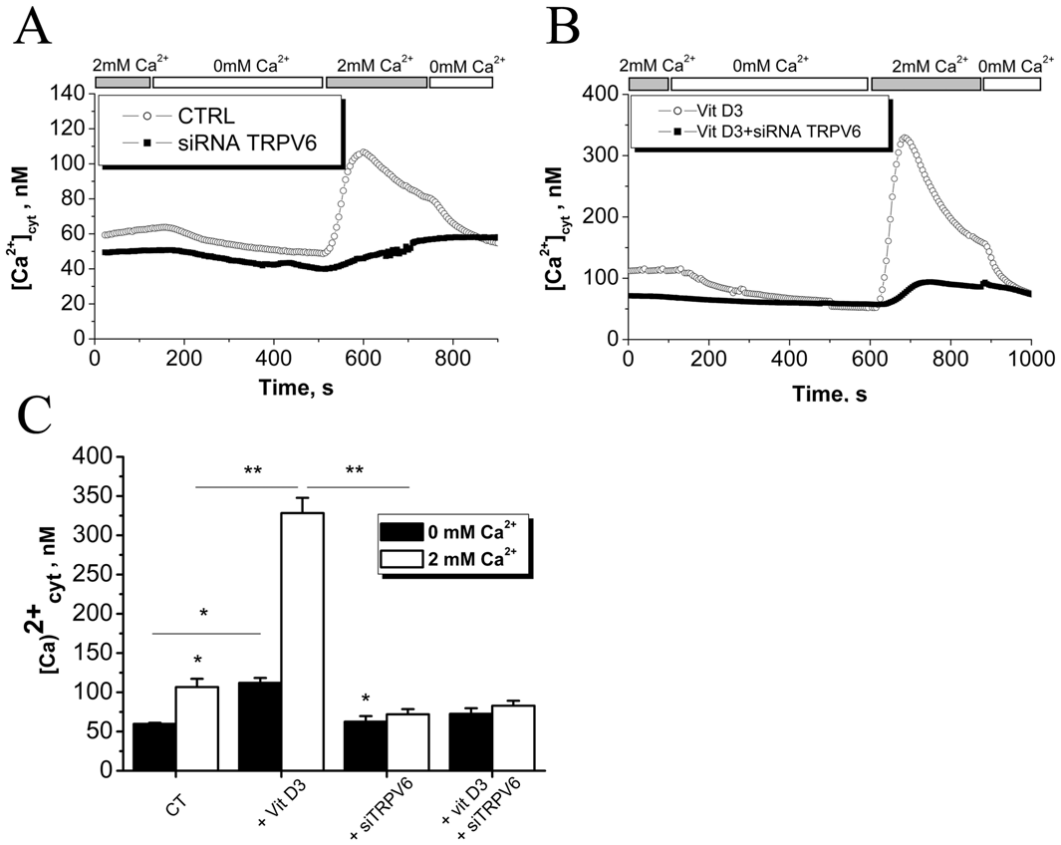
TRPV6 is an essential Ca^2+^-entry channel involved in 1,25-dihydroxyvitamin D3 increased Ca^2+^ uptake in LNCaP cells. **A**, TRPV6 involvement in Ca^2+^ uptake in LNCaP cells cultured in 2% FCS-containing RPMI medium and treated either with siCT (CTRL) or siRNA-TRPV6 (both 80 nM, 24 hours). **B**, Ca^2+^ uptake in LNCaP cells cultured in 2% FCS-containing RPMI medium and under 1,25-dihydroxyvitamin D3 (100 nM, 3 days) treated either with siCT (CTRL) or siRNA-TRPV6 (both 80 nM, 24 hours). **C**, a corresponding histogram showing relative [Ca^2+^]_i_ levels after consequent [Ca^2+^]_o_ switches in the conditions as indicated above. * - P<0.05 (as compared to control); ** - P<0.01, n = 140.

In order to study the contribution of TRPV6 as a highly Ca^2+^-selective channel to Ca^2+^-uptake in LNCaP cells, we measured intracellular calcium levels ([Ca^2+^]_i_) in LNCaP cells cultured in 2% FCS containing RPMI medium after consequent changes in extracellular calcium levels ([Ca^2+^]_o_). In control cells treated with EtOH (CTRL) the variation in [Ca^2+^]_o_ produced significant changes in [Ca^2+^]_i_ (Fig. 3A). siRNA-TRPV6 knockdown decreased the amplitude of 2 mM [Ca^2+^]_o_-evoked increase in [Ca^2+^]_i_ (Fig. 3A and C). 100 nM 1,25-dihydroxyvitamin D3 increased by itself basal [Ca^2+^]_i_ significantly as well as increased [Ca^2+^]_i_ response on application of 2 mM [Ca^2+^]_o_ which was completely reversed by the pretreatment with siRNA-TRPV6 (Fig. 3C). These data indicate that TRPV6 constitutively mediates Ca^2+^-uptake in LNCaP cells and TRPV6 also accounts for 1,25-dihydroxyvitamin D3-mediated enhanced Ca^2+^-uptake.

### The effects of 1,25-dihydroxyvitamin D3 on different androgen-independent cell lines

**Figure 4:**
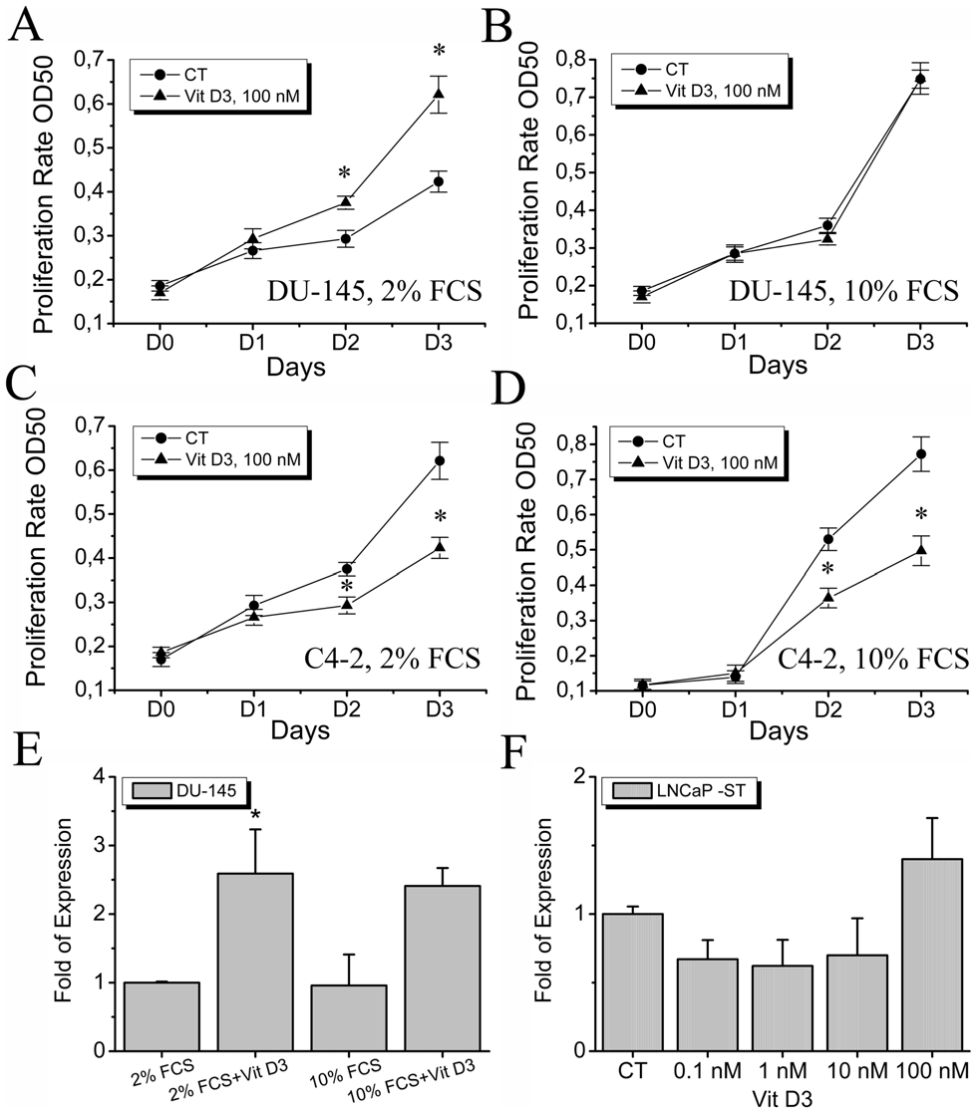
The effects of 1,25-dihydroxyvitamin D3 on androgen-independent cell lines. **A**, **B**, The effects of 1,25-dihydroxyvitamin D3 on androgen receptor-deficient DU-145 cell line in both 2 and 10% FCS-containing RPMI medium (**A** and **B**, respectively), * - P<0.05 (as compared to control), n = 3. **C, D**, The effects of 1,25-dihydroxyvitamin D3 on androgen-insensitive LNCaP C4-2 cell line in both 2 and 10% FCS-containing RPMI medium (**C** and **D**, respectively), * - P<0.05 (as compared to control), n = 3. **E**, the relative expression levels of TRPV6 channel in DU-145 cells treated with 100 µM 1,25-dihydroxyvitamin D3 for 3 days in 2 and 10% FCS-containing RPMI medium, * - P<0.05 (as compared to control), n = 3. **F**, the expression of TRPV6 channel induced by 100 nM 1,25-dihydroxyvitamin D3 for 3 days in LNCaP cells in steroid-deprived RPMI medium (LNCaP-ST), n = 3.

Two different androgen-independent cell lines were used: an androgen receptor-deficient DU-145 and androgen-insensitive LNCaP C4-2 cell lines. Cells were cultivated in the same conditions of 2 or 10% FCS supplemented RPMI medium and the effects of 1,25-dihydroxyvitamin D3 were studied (Fig. 4). The effects of 1,25-dihydroxyvitamin D3 on androgen receptor deficient DU-145 cell line were likely to be serum-dependent since in 2% FCS the proproliferative effects of 1,25-dihydroxyvitamin D3 were conserved (Fig 4A), whereas in 10% FCS its effects were abolished (Fig. 4B). The other cell line insensitive to steroids, but still expressing the androgen receptor, LNCaP C4-2 was used, where the effects of 1,25-dihydroxyvitamin D3 were shown to be FCS-independent and 100 nM 1,25-dihydroxyvitamin D3 exerted its strong anti-proliferative effects (Fig. 4C–D). A real time quantitative PCR was performed showing the regulation of TRPV6 expression in DU-145 cells by 100 µM 1,25-dihydroxyvitamin D3 in both 2 and 10% FCS containing medium (Fig. 4E). Steroid-deprived conditions in the case of LNCaP cells (LNCaP-ST) were also used to confirm that the induction of TRPV6 expression strongly depends on the steroid content of the culture medium (Fig. 4F). Thus the pro-proliferative effects of 1,25-dihydroxyvitamin D3 on the growth of PCa cells are determined by its ability to induce the expression of TRPV6 channel and its induction appears to be strongly steroid-dependent.

## Discussion

One of the most important finding of the present work is that 1,25-dihydroxyvitamin D3 may enhance proliferation of LNCaP cells. We have clearly shown that both proliferation rate and the number of the cells entering into the S-phase are increased upon 1,25-dihydroxyvitamin D3 treatment. These effects entirely depend on the expression and function of TRPV6 channel which has been previously shown to be implicated in prostate cancer growth and apoptosis-resistance [15]. A previously reported 1,25-dihydroxyvitamin D3 antiproliferative activity in prostate cancer may be compromised by TRPV6 upregulation.

A number of works has already published TRPV6 induction by 1,25-dihydroxyvitamin D3 in intestine [19], kidney [20], semicircular canal [21], and even prostate cancer cells [22]. Five VDR responsive elements were found in the human gene encoding the epithelial calcium channel TRPV6 suggesting its direct regulation by 1,25-dihydroxyvitamin D3 via its putative receptor [17]. We have confirmed in our cell model that the expression of TRPV6 is directly upregulated by 1,25-dihydroxyvitamin D3 in dose- and time-dependent fashion. Our results suggest that the nature of this upregulation is steroid-dependent since in steroid-deprived conditions the effects of 25-dihydroxyvitamin D3 are abolished. This finding is consistent with the data that activities of 1,25-dihydroxyvitamin D3 in LNCaP cells are dependent upon steroid co-regulation and that, for instance, androgen receptor upregulation by 1,25-dihydroxyvitamin D3 likely contributes to the synergistic actions of 1,25-dihydroxyvitamin D3 and DHT in these cells [23]. The data from the laboratory of Feldman show that the addition of DHT at 1 nM to the medium restored the antiproliferative activity of 1,25-dihydroxyvitamin D3, whereas an antiandrogen, Casodex, completely blocked 1,25-dihydroxyvitamin D3 antiproliferative and PSA stimulation activities when cells were cultured in FBS medium [23].

The ability of 1,25-dihydroxyvitamin D3 to inhibit prostate growth has been demonstrated in primary cultured cells from normal tissues, benign prostatic hyperplasia (BPH) and prostate cancer, and several xenograft models of prostate cancer [5], however, no relation to TRPV6 responsiveness has been demonstrated so far. The mechanism for 1,25-dihydroxyvitamin D3 activity is not completely clear but relates to different activities as to pre-receptor differences in pharmacokinetics, as well as differences in the functional conformation of the ligand-bound VDR complex which can alter properties of retinoid X-receptor hybridization, DNA binding and co-activator recruitment [24]. The mechanism of growth inhibition by 1,25-dihydroxyvitamin D3 appears to be mutifactorial but induction of p21^WAF1/CIP1^ and/or p27^Kip1^ seems to be a major pathway [25].

We are the first to report that the effects of 1,25-dihydroxyvitamin D3 may be pro-proliferative when mediated by the direct induction of *trpv6* gene expression in human highly cancerous androgen-dependent LNCaP cell line. The question remains open whether 1,25-dihydroxyvitamin D3 treatment is feasible in cancer stages and metastasis being distinct in high TRPV6 expression, or, otherwise, in the prostate cancer cells biopsies still responsive to 1,25-dihydroxyvitamin D3 treatment by overexpressing TRPV6.

Thus, 1,25-dihydroxyvitamin D3 upregulates TRPV6 which considerably increases [Ca^2+^]_i_ providing enhanced Ca^2+^-uptake by LNCaP cells. This 1,25-dihydroxyvitamin D3-induced Ca^2+^-uptake dramatically increases proliferation rate and a number of the cells entering into the S-phase and also contributes to the enhanced apoptosis resistance. Intriguingly, the apoptosis remains unaffected upon 1,25-dihydroxyvitamin D3 treatment which may be explained by the responsiveness of LNCaP cell line to 1,25-dihydroxyvitamin D3 via increasing the expression of TRPV6 channel and therefore enhancing the resistance to apoptosis. However, when LNCaP cells are treated with 1,25-dihydroxyvitamin D3 but pretransfected with siRNA-TRPV6 and thus void of this channel they are much more subjected to apoptosis that it becomes comparable to impact of siRNA against AR used a positive control. This implies that the calcium supplied into the cancer cell via TRPV6 channel is used to counteract the effects of 1,25-dihydroxyvitamin D3 which have to be antiproliferative in the absence or low presence of this channel. We conclude that TRPV6 is a serious determinant for 1,25-dihydroxyvitamin D3 pro- or antiproliferative activity.

Our data are not contradictory to the previously published works and are consistent with the hypothesis that the growth inhibitory effects of 1,25-dihydroxyvitamin D3 are partially mediated through its ability to modulate PCNA expression [26]. A PCNA protein level being two-fold decreased upon 1,25-dihydroxyvitamin D3 treatment is further declined in LNCaP cells transfected with siRNA-TRPV6, with or without 1,25-dihydroxyvitamin D3. These conditions are characterized by the suppression of cell proliferation, therefore suggesting a potent contramechanism mediated by TRPV6.

1,25-dihydroxyvitamin D3 also up-regulates the expression of androgen receptor (AR) and PSA, and both biochemical and immunohistochemical analyses show proportionately greater increased presence of AR in the nucleus and reduced in the cytosol [27]. These evidences allow to suggest multivectorial differential effects of 1,25-dihydroxyvitamin D3 on the proliferation machinery, especially in cancer. A two-fold downregulation of a particular set of DNA replication genes including a cell division cycle 6 homolog, a DNA polymerase alpha subunit, PCNA, two DNA polymerase delta subunits, and flap-structure specific endonuclease 1 [28], seems unlikely to drastically affect proliferation by itself.

On the other side, 1,25-dihydroxyvitamin D3 is known to stimulate DNA synthesis via sequential activation of Raf and the mitogen-activated protein kinase [29]. VDR protein was also shown to associate with Shc, indicating that this steroid hormone is able to signal through the transcription-independent pathways similar to those used by peptide hormones and cytokines [29]. A combination of 1,25-dihydroxyvitamin D3 and DHT has already been demonstrated to increase DNA synthesis in LNCaP cells [30], however the other works show either no significant or inhibition of proliferation by 1,25-dihydroxyvitamin D3 alone. Moreover, 1,25-dihydroxyvitamin D3 stimulates the proliferation of vascular smooth muscle cells [31], epiphyseal chondrocytes [32], myoblasts [33], skin cells [29], [34], mammalian epithelial cells [35], myeloid leukemia cell lines HL-60 and KG-1a [36], T-cells of tumor bearers [37], chromaffin cells [38], carcinoma C-cells [39] etc.

The genomic sequence corresponding to 6000 bp upstream and 100 bp downstream of hTRPV6 ATG has been taken for the transcription factor analysis (data not shown). The MatInspector 7.7.3 program (Genomatix Software GmbH) has been employed to analyse the putative steroid receptor binding sites [40]. The hTRPV6 promoter sequence was analysed for the presence of different steroid-responsive elements using prostate specific matrix which is associated with transcription factors expressed and transcriptionally active in this tissue. Numerous steroid-responsive elements including but not limited to VDR, androgen receptor, and glucocorticoid receptors, were found which suggests the possible strong regulation of *trpv6* gene by different steroid receptors and therefore may represent certain temporal and spatial limits for each particular nuclear receptor to induce the transcription of the *trpv6* gene. This latter evidence may explain why in high level steroid conditions the expression of TRPV6 channel may be not affected or even downregulated which may trigger the activation of a different pathway than expected.

The combined 1,25-dihydroxyvitamin D3 therapy has recently become an advantage in treating prostate cancer. The combined treatment with other compounds interacting directly or indirectly with the VDR pathway like inhibitors of histone deacetylation [41], a non-steroid anti-inflammatory drug [42], or genistein and trichostatin A [43], will shift downstream signaling to the required direction to achieve beneficial effects.

In conclusion, we have shown that TRPV6 is directly implicated in 1,25-dihydroxyvitamin D3-stimulated proliferation in low steroid conditions. The apoptosis resistance due to TRPV6 channel may be overcome by synergistic action of 1,25-dihydroxyvitamin D3 and selective TRPV6 knockdown. 1,25-dihydroxyvitamin D3-induction of TRPV6 expression should be taken into account while treating TRPV6-positive/inducible tumors. The data strongly suggest that the ability of 1,25-dihydroxyvitamin D3 to induce the expression of TRPV6 channel is the crucial determinant of the success or failure of 1,25-dihydroxyvitamin D3-based therapies.
